# Chronic Obstructive Pulmonary Disease (COPD) and Lung Cancer: Epithelial Mesenchymal Transition (EMT), the Missing Link?

**DOI:** 10.1016/j.ebiom.2015.10.016

**Published:** 2015-10-17

**Authors:** Sukhwinder Singh Sohal

**Affiliations:** School of Health Sciences, University of Tasmania, Launceston, Tasmania 7250, Australia

Lung cancer and chronic obstructive pulmonary disease (COPD) commonly coexist in smokers, and the presence of COPD increases the risk of developing lung cancer by 4–5 folds, even when the smoking history is controlled for (Sohal, SS, et al., 2014a, Sohal, S, et al., 2013). Lung cancer may consist of small cell carcinoma and non-small cell carcinomas encompassing squamous cell carcinoma, adenocarcinoma and large cell carcinoma. In a recent very comprehensive study by Huang and colleagues published in *EBioMedicine*, provided compelling evidence suggesting strong association between smoking, COPD and small cell lung cancer (SCLC) (Huang et al., 2015). This publication is a very timely reminder highlighting the importance of this lethal association between COPD/lung cancer and warrants further experimental studies exploring this link not only in SCLC but lung cancer in general. Unfortunately, the research effort directed into this has been disproportionately weak compared to its clinical and scientific importance, and indeed COPD itself is the least researched of all common chronic conditions compared to its social importance.

Lung cancer and COPD share a common etiology i.e. mainly tobacco smoking. This implies that mechanisms specific to COPD may be involved in the development of lung cancer (Sohal, S, et al., 2013, Yang, IA, et al., 2011). Potential shared biological mechanisms in COPD and lung cancer include: chronic inflammation, matrix degradation, cell proliferation and anti-apoptosis, abnormal wound repair and angiogenesis, but perhaps especially the process of epithelial mesenchymal transition (EMT), a highly plastic process in which epithelial cells change into a mesenchymal phenotype (Yang et al., 2011). There is much support for a major role for EMT in the pathogenesis of epithelial cancers (Garber, 2008). It is clear that several of the key pathways driving EMT, which is designed teleologically for the highly controlled process of embryogenesis, are also aberrantly activated in cancer (Sohal et al., 2014a). EMT is a vital process during embryogenesis (Type I EMT), but can also be induced as a result of persistent damage and inflammation. There are then two subsequent outcome possibilities with active EMT: severe and even complete organ fibrosis and secondarily association with malignancy; both of these processes may be relevant to COPD pathology and natural history. Lung cancer evolves as a result of a series of mutational events in epithelial cells that have been studied in detail by numerous investigators. However, it is not only epithelial malignant transformation that matters, but also the environment in which these cells develop i.e. the tumor microenvironment. We suggest that EMT-Type-3 provides a pro-cancer stroma that both stimulates epithelial cancer cell transformation, in addition to the genetic changes induced by cigarette smoke, but then allows these cells to thrive, invade and metastasise (Mahmood et al., 2015). In a separate twist to this complex story, the epithelial cancer itself also directly uses EMT mechanisms to spread and metastasise.

It is of interest and relevance in this context that over 90% of human cancer arises in epithelia (e.g., breast, colon, stomach, liver, prostate, ovary/fallopian tube and bladder), and the involvement of EMT in all of these may be a central paradigm (Garber, 2008). It is pertinent that up to 70% of lung cancer occurs in the context of mild-to-moderate (not severe) COPD (Barnes and Adcock, 2011), and COPD-related cancer may well be just another example of this core principle of unstable epithelium in the context of tissue inflammation and/or chronic stimulation.

Recently, we reported (Sohal et al., 2014b) in a randomized controlled trial that inhaled corticosteroid fluticasone propionate given over six months suppressed EMT-related changes in large airways of COPD patients. This trial showed marked reduction in EMT related changes in the active inhaled corticosteroids (ICS) compared to placebo (Sohal et al., 2014b). This is the first study reporting anti-EMT effects of inhaled corticosteroids in COPD, where of course they are widely used as putative “anti-inflammatory” agent, although that mode of operation is far from convincing. In human epidemiological studies it is strongly suggested that patients on inhaled corticosteroids, albeit only at high doses (as used in our study), are associated with an appreciable (50%) reduction in the risk of lung cancer (Parimon et al., 2007). However, the TORCH study (Calverley et al., 2007) of ICS in severe COPD failed to show this effect, but it was not powered to pick this up, and was a study on severe rather than mild to moderate COPD where lung cancer particularly occurs (Barnes and Adcock, 2011).

We suggest that EMT might be the process by which this effect of ICS occurs. If this is true, it has huge implications for therapeutic and public health policy, since it is strongly suggested in literature that patients on ICS are associated with a decreased risk for lung cancer (Parimon et al., 2007). It is suggested that statins may also have similar effects on EMT in COPD, since lung cancer risk decreases in COPD patients who are on statins (Sohal, SS, et al., 2014a, Sohal, S, et al., 2013), this warrants further studies. There is an essential need for both in vivo and in vitro human studies to understand the mechanistic link between COPD and airway cancer and how ICS and the other drugs may affect it. Only by understanding what is happening and the mechanism involved, will new therapeutic possibilities emerge.

## Disclosure

The author declared no conflicts of interest.
